# Comprehensive analysis to identify a novel diagnostic marker of lung adenocarcinoma and its immune infiltration landscape

**DOI:** 10.3389/fonc.2023.1199608

**Published:** 2023-06-20

**Authors:** Ankang Zhu, Dongchen Pei, Yan Zong, Yan Fan, Shuai Wei, Zhisong Xing, Shuailin Song, Xin Wang, Xingcai Gao

**Affiliations:** ^1^ The Fifth Affiliated Hospital of Zhengzhou University, Zhengzhou University, Zhengzhou, China; ^2^ Department of Cardiothoracic Surgery, The Fifth Affiliated Hospital of Zhengzhou University, Zhengzhou University, Zhengzhou, China

**Keywords:** lung adenocarcinoma, immune cell infiltration, biomarkers, immune-related pathways, LASSO, RT-qPCR

## Abstract

**Background:**

Lung cancer continues to be a problem faced by all of humanity. It is the cancer with the highest morbidity and mortality in the world, and the most common histological type of lung cancer is lung adenocarcinoma (LUAD), accounting for about 40% of lung malignant tumors. This study was conducted to discuss and explore the immune-related biomarkers and pathways during the development and progression of LUAD and their relationship with immunocyte infiltration.

**Methods:**

The cohorts of data used in this study were downloaded from the Gene Expression Complex (GEO) database and the Cancer Genome Atlas Program (TCGA) database. Through the analysis of differential expression analysis, weighted gene co-expression network analysis (WGCNA), and least absolute shrinkage and selection operator(LASSO), selecting the module with the highest correlation with LUAD progression, and then the HUB gene was further determined. The Gene Ontology (GO), Kyoto Encyclopedia of Genes and Genomes (KEGG), and Gene Set Enrichment Analysis (GSEA) were then used to study the function of these genes. Single-sample GSEA (ssGSEA) analysis was used to investigate the penetration of 28 immunocytes and their relationship with HUB genes. Finally, the receiver operating characteristic curve (ROC) was used to evaluate these HUB genes accurately to diagnose LUAD. In addition, additional cohorts were used for external validation. Based on the TCGA database, the effect of the HUB genes on the prognosis of LUAD patients was assessed using the Kaplan-Meier curve. The mRNA levels of some HUB genes in cancer cells and normal cells were analyzed by reverse transcription-quantitative polymerase chain reaction (RT-qPCR).

**Results:**

The turquoise module with the highest correlation with LUAD was identified among the seven modules obtained with WGCNA. Three hundred fifty-four differential genes were chosen. After LASSO analysis, 12 HUB genes were chosen as candidate biomarkers for LUAD expression. According to the immune infiltration results, CD4 + T cells, B cells, and NK cells were high in LUAD sample tissue. The ROC curve showed that all 12 HUB genes had a high diagnostic value. Finally, the functional enrichment analysis suggested that the HUB gene is mainly related to inflammatory and immune responses. According to the RT-qPCR study, we found that the expression of DPYSL2, OCIAD2, and FABP4 in A549 was higher than BEAS-2B. The expression content of DPYSL2 was lower in H1299 than in BEAS-2B. However, the expression difference of FABP4 and OCIAD2 genes in H1299 lung cancer cells was insignificant, but both showed a trend of increase.

**Conclusions:**

The mechanism of LUAD pathogenesis and progression is closely linked to T cells, B cells, and monocytes. 12 HUB genes(ADAMTS8, CD36, DPYSL2, FABP4, FGFR4, HBA2, OCIAD2, PARP1, PLEKHH2, STX11, TCF21, TNNC1) may participate in the progression of LUAD *via* immune-related signaling pathways.

## 1 Introduction

Lung cancer is one of the most common cancers in the world. In recent years, the number of new cases has reached a peak, and lung cancer has the highest number of deaths (1, 2). Lung adenocarcinoma has the highest incidence of lung cancer at approximately 40% (3). While one of the reasons for the high mortality of LUAD is that 57% of cases had progressed at the time of testing, when the treatment regimen was limited, with 1-and 5-year survival rates of only 26% and 4%, respectively (4, 5). This result is not satisfactory, and although the rapid development of immunotherapy and targeted therapies in recent years has led to a significant improvement in the outcomes of LUAD patients, the prognosis outcome of LUAD patients is still unsatisfactory (6, 7). Thus, there is a need to investigate and discover novel biomarkers or immune cell infiltration during LUAD progression, which is of extraordinary importance for the early detection, diagnosis, treatment, and better prognosis of LUAD. Despite the diverse pathogenesis and causes of LUAD, extensive clinical evidence and experimental data show that immunocytes and immune-related pathways play various roles in the development of LUAD and the prognostic process. For example, a reduction in CD4 + T cells suppresses the activity of cytotoxic T cells in tumors, thereby restricting LUAD tumor cell growth (6). Programmed cell death 1 (PD-1) is expressed in T cells to suppress peripheral autoimmunity (immune tolerance) (8). M2-polarized macrophages exhibit immunosuppressive activity and promote tumor angiogenesis in LUAD patients (9). Many other molecules are closely associated with LUAD and play an immunological role in tumor progression. Thus, further investigation into the molecular mechanisms of LUAD pathogenesis is still needed.

WGCNA works by analyzing a large number of genes and then putting genes with similar expressions into the same module according to the clustering principle. The most significant advantage of this method over simple cluster analysis is that it is biologically meaningful and allows for effective preliminary screening of genes related to target features (10, 11). In many cases, LASSO algorithms are used to describe the degree of correlation between two related variables. The advantage of this algorithm over the traditional Cox regression and logistic regression lies in its ability to reduce the dimension. Both WGCNA and LASSO regression analysis are commonly used for bioinformatics technology analysis. Moreover, the LASSO analysis of the WGCNA genes can make us more accurate in screening the target feature-related genes (12). In the first step, we screened differentially expressed genes and identified key biomarkers for LUAD progression. Based on the results of the Gene Ontology (GO) of differentially expressed genes and the Kyoto Encyclopedia of Genes and Genomes (KEGG), we found that these DEGs mainly focus on some immune processes and immune pathways related to LUAD. We then used ssGSEA analysis to assess the infiltration of immunocytes in the immune environment in the hope of gaining a clearer understanding of the mechanisms of LUAD progression, and the results may provide a way to understand the pathogenesis of LUAD and find new therapeutic targets.

## 2 Materials and methods

### 2.1 Data collection

Microarray expression data and clinical information for LUAD were obtained from the GEO and TCGA databases. There were two cohorts in the treatment group, GSE63459 and GSE176348, with 89 specimens (including 45 tumor samples and 44 normal samples). In addition, external validation using the TCGA-LUAD cohort with 598 samples (including 539 tumor samples and 59 normal samples) was performed. All sequencing information for normal samples comes from adjacent tissues.

### 2.2 Selection of the DEGs

We used the data normalization and probe annotation from the R software (version 4.2.1) “limma” and “GEOquery” packages for the data of GSE63459 and GSE176348, with P-value < 0.05 and |log fold change (FC) | > 1 for the DEGS screening criteria (13, 14).

### 2.3 Construction of gene co-expression network

We used the WGCNA to process expression profile data from GSE63459 and GSE176348 datasets to establish a weighted co-expression network. Then we investigated the genes that deviate from the top 25% of the median (10). The data integrity is checked by the ‘Good SampleGenes’ function. We chose a suitable soft threshold value (β) and validated the ability of the soft threshold value. The matrix data was transformed into an adjacency matrix by us, followed by clustering to identify modules based on the topological overlap. Then, the module feature element (ME) is calculated, the similarly expressed modules are combined into the cluster tree according to the ME, and we draw the hierarchical cluster tree graph. Then, the module and phenotype data are combined, and then the gene significant (GS) and module significant (MS) are calculated; the calculation results are used to evaluate whether the gene and clinical information are essential and to analyze the correlation between the module and the model. In addition, We calculated the module membership (MM) for each gene to analyze the GS values of each module.

### 2.4 Selection and validation of the HUB genes

The gene with the highest inter-module connectivity was selected as the candidate HUB gene. The GS values for biologically significant genes are also generally higher. Therefore, we chose candidate genes with an absolute GS value> 0.20 and an MM value > 0.80. We then intersected these genes with DEGS using the “glmnet” package in the R software package and used the LASSO analysis to determine the final HUB genes (11). We used box plots to probe the HUB gene expression levels in LUAD samples and healthy samples. With the help of ‘MedCalc’ software (version 2.0.1), we draw the receiver operating characteristic curves (ROC) to determine these HUB genes’ diagnostic specificity and accuracy. A dataset (TCGA-LUAD) is also available for external verification of the HUB gene’s expression level and diagnostic value.

### 2.5 Prognostic analysis

With the help of the “Survival” and “SurvMiner” packages in the “R” software, we divided the samples in TCGA-LUAD into two groups (high and low expression groups) using the median expression of the HUB gene. Lastly, survival curves for HUB and LUAD genes were plotted using the Kaplan-Meier method with the aid of the software package “ggplot2”.

### 2.6 Immunohistochemical staining was performed

Results of immunohistochemical staining of the HUB gene in normal lung tissue and lung cancer specimens from The Human Protein Atlas(www.proteinatlas.org).

### 2.7 Assessment of immunocytes infiltration and its association with HUB genes

We quantified the infiltration of 28 immunocytes in the GSE63459 and GSE176348 datasets using the ssGSEA algorithm (15). The box plots we established indicate the differences in the expression levels of these immune cells. We also calculated the Spearman correlation of these immune-infiltrating cells with the candidate HUB genes and visualized the calculated results with the ‘ggplot2’ program package.

### 2.8 Functional enrichment analysis

We performed GO analysis of DEGs, KEGG analysis, and GSEA analysis through the ‘clusterProfiler’ and ‘enrichplot’ package of the R software package (16). We used the immunological signature genomes from the Molecular Signature Database (MsigDB) as the reference, and the significantly enriched genomes had to meet the P <0.05 and the false discovery rate (FDR) q-value <0.05.

### 2.9 Experimental validation

Several HUB genes (DPYSL2, OCIAD2, and FABP4) were selected for study to verify the HUB genes’ role further. Normal human lung epithelial cells (BEAS-2B) and lung adenocarcinoma cells (A549 and H1299) were collected for culture, moreover, extracted RNA from the three cells using the Trizol reagent. For cDNA synthesis, the synthesis was performed using the reverse transcription reagent VAZYME R232. The final PCR reaction was performed on a quantitative real-time PCR instrument. The reaction parameters included the denaturation process (30s at 95°C), followed by 40 PCR cycles (5s at 95°C and 34s at 60°C). The melting curve of the PCR product was established, and after the amplification reaction, the temperature was slowly heated from 60°C at (95°C, 15s, 60°C, 60s, 95°C, 15 s) to 99°C (instrument automatic ramp rate 0.05°C/s). Quantitative real-time PCR reactions were performed for target and housekeeping genes for each sample. We calculated the relative expression levels of the three genes using the 2 ^ (-δδ ct) method. Since the experimental results of FABP 4 and DPYSL2 were fit to a normal distribution, the analysis was performed using the one-way ANOVA test. We used the Kruskal-Wallis test for statistical analysis for OCIAD2 genes whose results do not conform to the normal distribution (**Supplementary file 1**). The sequence of the primers is as follows:

DPYSL2, 5’-CCCTGCAGAACATCAACGAC-3’ (forward) and 5’-GGCATCTGGAAACGAGTGTG-3’ (reverse); OCIAD2, 5’-GTCTGCTCGTGGAAACCAAG-3’ (forward) and 5’-CAAGAGACCAGCAAGTGCAAC-3’ (reverse); FABP4, 5-GATGACAGGAAAGTCAAGAGCAC-3’ (forward) and 5’-GACGCCTTTCATGACGCATTC-3’ (reverse); and GADPH,5’-TCTGACTTCAACAGCGACACC-3’ (forward) and 5’-CTGTTGCTGTAGCCAAATTCGT-3’ (reverse).

## 3 Results

### 3.1 Establishment of a co-expression network and selection of key modules

**Figure 1:**
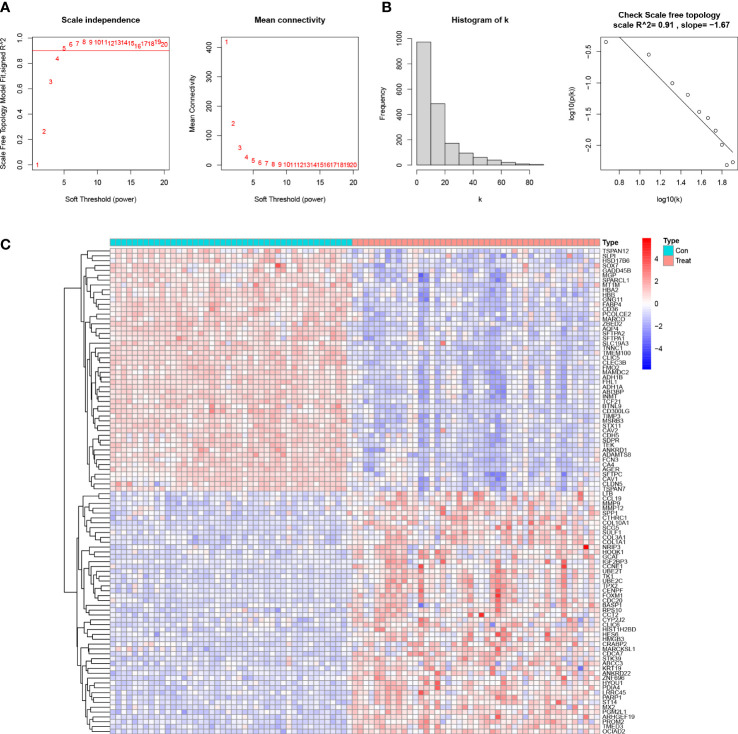
Determine the soft threshold ability in WGCNA. **(A)** Scale-free fit index and average connectivity for different soft threshold powers (β). Positions with a correlation coefficient of 0.9 are marked with a red line, corresponding to a soft threshold power of 5. **(B)** Histogram of the connectivity distribution and checking the scale-free topology map. **(C)** Heatmap of the correlation of genes in the experimental and control groups.

**Figure 2:**
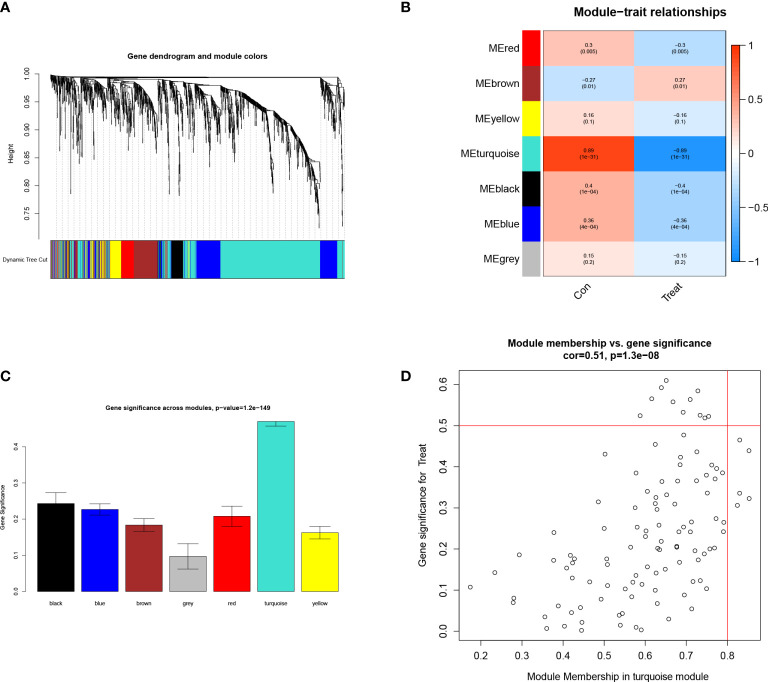
Establishment of the WGCNA module. **(A)** Cluster plot of genes in the top 25% of the median absolute deviation. Each color in the horizontal axis corresponds to a module, and each branch in the graph indicates the gene. **(B)** Heat map of the module-characteristic relationship. **(C)** Bar graph of the distribution of average gene significance in different modules. **(D)** Scatterplot depicting the relationship between gene module membership and gene significance in the turquoise module.

The absolute deviations in the median top 25% of genes were selected for constructing WGCNA, and missing values and outliers in the samples were subsequently removed by cluster analysis. To maintain consistency with the scale-free network, we set the soft threshold β to 5 (scale-free R2 = 0.91; slope =-1.67) (**Figures 1A, B**). We also analyzed the gene expression in the normal and LUAD samples and plotted the results as a heatmap(**Figure 1C**). We built a co-expression matrix and obtained seven modules with the help of dynamic hybridization shear (**Figure 2A**). The relationship between these seven modules and the LUAD and healthy samples is shown in the heatmap. The turquoise module has the highest correlation (cor) (cor = 0.89; P=1e-31) (**Figures 2B, C**). Moreover, after our correlation analysis, we found that in the turquoise module, GS and MM are also well correlated(cor=1.51; P=1.3e-08) (**Figure 2D**).

### 3.2 Identification of DEGs and selection of HUB genes

**Figure 3:**
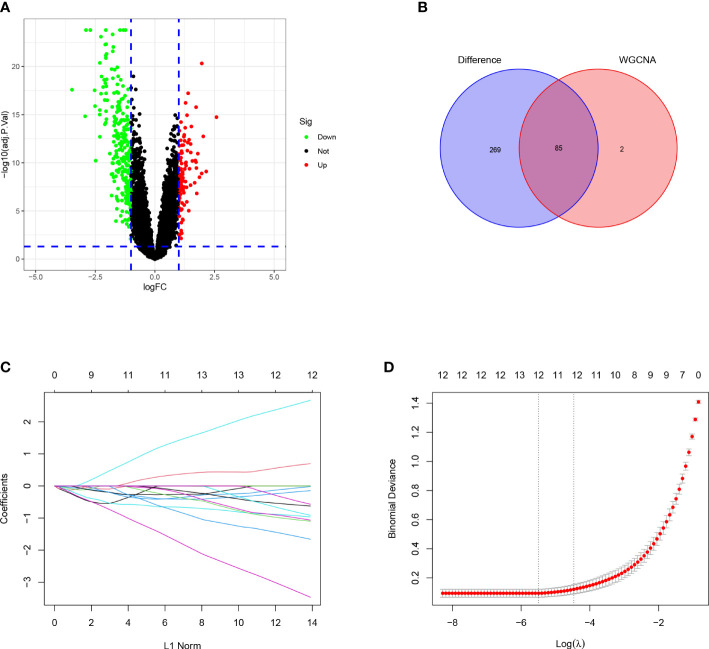
Identification of the DEGS and selection of the HUB genes. **(A)** Volcano plot of DEGS between LUAD samples and healthy control tissues. **(B)** A Venn diagram of the intersection of the DEGS and the turquoise module. **(C)** The relationship of partial likelihood bias with log **(L)** changes plotted by LASSO regression in the 10-time cross-validation. **(D)** Distribution of LASSO coefficient for 12 HUB genes in 10-fold cross-validation.

For the DEGs, our filtering criteria were P <0.05 and | logFC |> 1, including 354 differential expressed genes and displaying the results on the volcano plot (**Figure 3A**). We further selected 87 genes with higher connectivity in the turquoise module using |GS|> 0.20 and |MM|> 0.80 as screening criteria. The results of these two screens were compared, and their intersection was selected as candidate HUB genes, and 85 genes were combined (**Figure 3B**). Ultimately, after a further screening of these genes using the LASSO analysis, we were able to obtain 12 genes (ADAMTS8, CD36, DPYSL2, FABP4, FGFR4, HBA2, OCIAD2, PARP1, PLEKHH2, STX11, TCF21, TNNC1) (**Figures 3C, D**).

### 3.3 Functional enrichment analysis of DEGs

**Figure 4:**
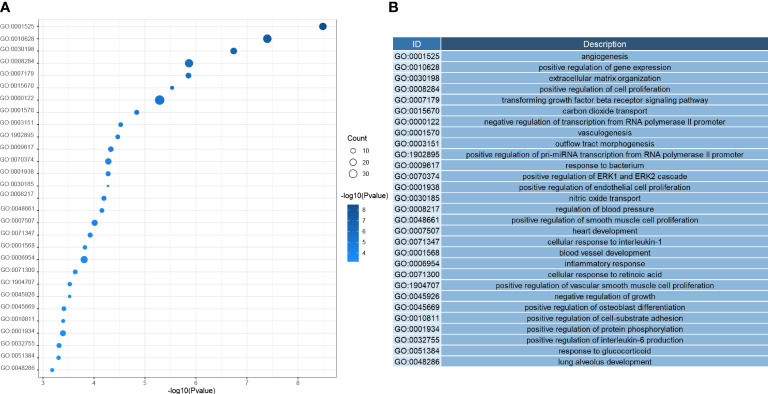
**(A)** Heatmap of biological process enrichment of DEGs. **(B)** Corresponding annotation for the GO ID.

**Figure 5:**
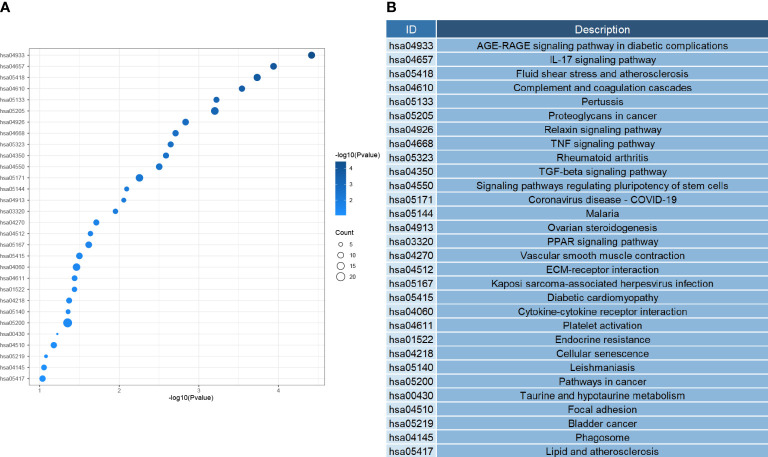
**(A)** Heatmap of signal pathway enrichment of DEGs. **(B)** The KEGG ID corresponds to the annotation.

Next, we investigated the function of the DEGs screened above; We performed GO analysis on 354 genes. According to the results, we know that DEGs mainly focus on the regulation of genes or pathways (e.g., transforming growth factor receptor signaling pathway, positive regulation of gene expression, negative regulation of transcription by RNA polymerase II promoter), vascular development (e. g., angiogenesis, angiogenesis, vascular development), immune response and inflammatory response (e. g. inflammation, cellular response of interleukin-1, positive regulation of interleukin-6 production), and even play an essential role in alveolar development (**Figure 4**). According to the KEGG analysis, We can also learn that DEGs are mainly enriched in the following pathways, pathways of immunological and inflammation-related diseases (AGE-RAGE signaling pathway in diabetic complications, fluid shear stress, and atherosclerosis, rheumatoid arthritis), there are also some immune-related pathways (IL-17 signaling pathway, TNF signal channel, the TGF signaling pathway, PPAR signaling pathway, and other pathways)(**Figure 5**). The GO analysis and KEGG analysis showed that there are many biological processes and signaling pathways related to the immune-inflammatory response in the development and development of LUAD.

### 3.4 Immunohistochemical staining of HUB genes in normal tissues and tumor tissues

**Figure 6:**
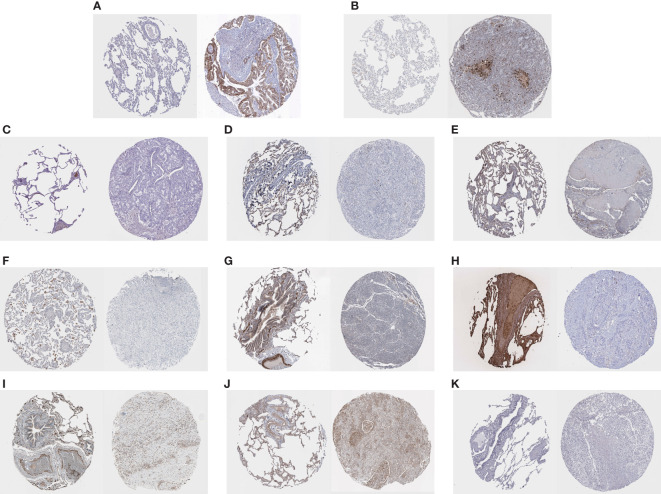
**(A–K)** Results of the immunohistochemical staining of OCIAD2, PARP 1, ADAMTS8, CD36, DPYSL2, FABP4, FGFR4, HBA2, STX11, TCF21, and TNNC 1, on the left of each pair of images, is the staining of genes in normal lung tissue, and on the right is the staining in lung cancer samples.

IHC staining results were paired as shown in **Figure 6**. On the left of each pair of images is the gene staining in normal lung tissue, and on the right is the staining in lung cancer samples. We can estimate the expression level of HUB genes, and it is clear that two HUB genes, OCIAD2 and PARP 1, have higher expression in lung cancer samples (**Figures 6A, B**), while the remaining HUB genes have more expression in normal samples (**Figures 6C–K**). Unfortunately, we were unable to find the PLEKHH2 staining results, and we will continue to follow up on the results in follow-up studies.

### 3.5 Validation of HUB gene expression levels and diagnostic value

**Figure 7:**
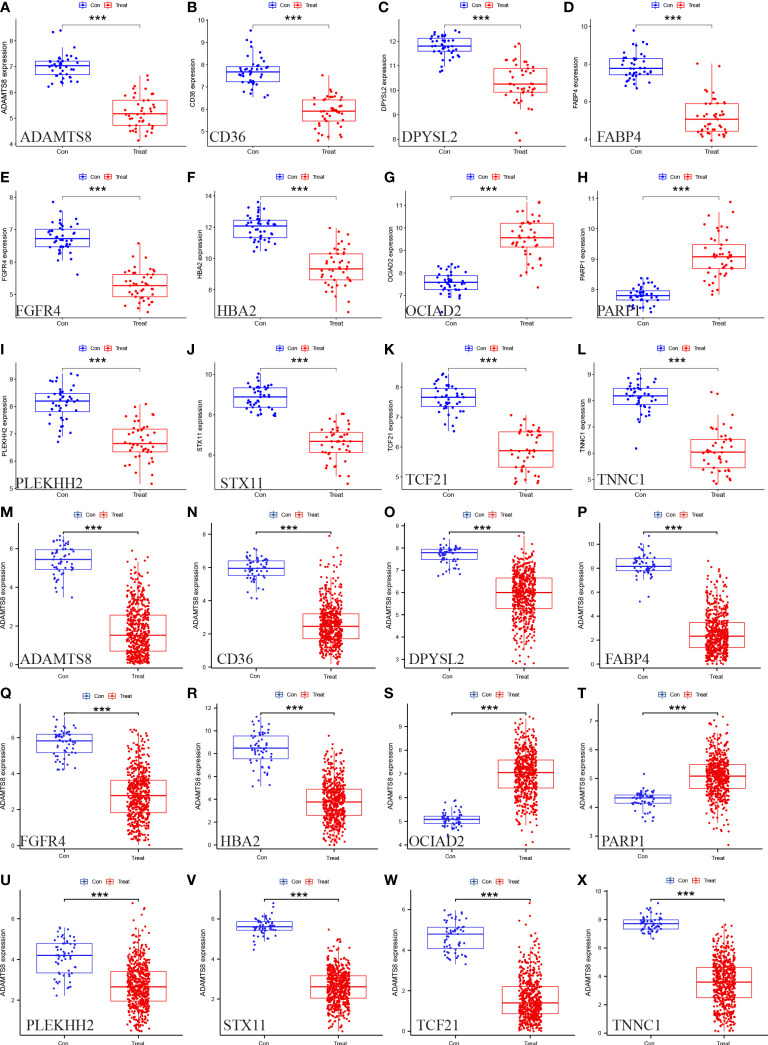
Verification of the 12 HUB genes at the gene expression level. **(A–L)** Verification of the HUB genes in the GSE63459 and GSE176348 **(M–X)** Verification of the HUB gene in the TCGA-LUAD cohort (* represents P <0.05, ** represents P <0.01, and *** represents P <0.001).

**Figure 8:**
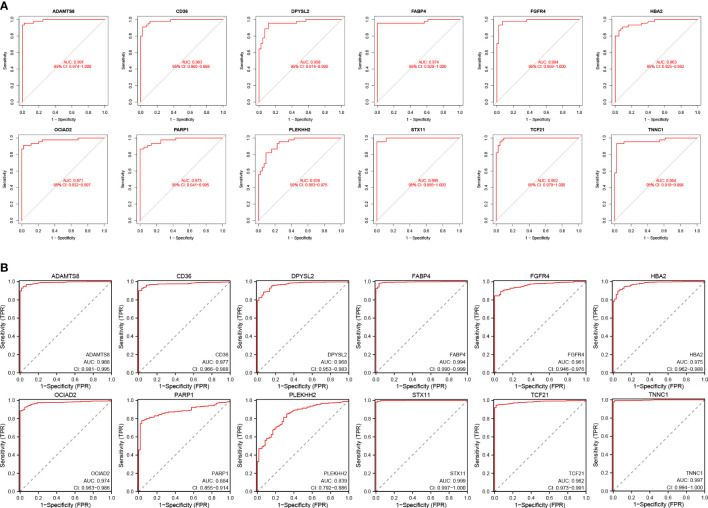
Verification of the diagnostic value of the 12 HUB genes. **(A)** Verification of the HUB genes in the GSE63459 and GSE176348 cohorts. **(B)** Validation of the HUB gene in the TCGA-LUAD cohort.

We assessed the expression levels of the 12 HUB genes by box plots. The results indicated that only OCIAD2, PARP 1 were significantly increased in the control group (**Figures 7G, H**) (P <0.001), while the other ten genes, ADAMTS8, CD36, DPYSL2, FABP4, FGFR4, HBA2, PLEKHH2, STX11, TCF21, TNNC1 were higher in the control group (**Figures 7A–F, I–L**). Next, we also externally verified the expression levels of these 12 HUB genes using the TCGA-LUAD dataset, and validation results were in agreement with our experimental group (**Figures 7M–X**). In the ROC curve analysis of the 12 HUB genes, the area under the curve (AUC) of the HUB gene represents the sensitivity and specificity of the HUB gene for the diagnosis of LUAD. From the ROC curve, we can know that the AUC values of all 12 HUB genes were> 0.93, indicating the high value of HUB genes for the diagnosis of LUAD (**Figure 8A**). While in the TCGA-LUAD cohort, the AUC values, except for PAPR 1 and PLEKHH2, were 0.884 and 0.839, respectively. The AUC values for the remaining HUB genes were all> 0.95, which coincident with the findings from our cohort study above (**Figure 8B**).

### 3.6 Prognostic analysis of HUB genes

We partitioned LUAD samples into two groups (high and low expression groups) based on the TCGA-LUAD database. Kaplan-Meier curves were performed for the HUB gene in order to analyze its prognostic relationship to LUAD patients. According to the results, the high expression of OCIAD2 and PARP1 is linked to poor prognosis in LUAD patients (**Supplementary Figures 1G, H**). However, high expression of ADAMTS8, CD36, DPYSL2, PLEKHH2, STX11, and TCF21 tends to lead to better prognosis (**Supplementary Figures 1A–F, I–L**), which coincides with the difference in expression of these HUB genes in normal samples and lung cancer samples.

### 3.7 Immune cell infiltration and its correlation with HUB genes

**Figure 9:**
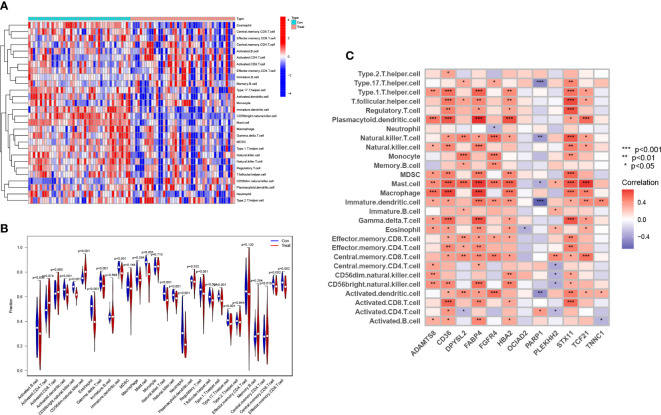
Analysis of the immune microenvironment associated with LUAD. Both **(A)** and **(B)** show the distribution of 28 immune cells in the immune microenvironment of normal and LUAD samples. **(C)** The relationship of the 12 HUB genes with the infiltration of multiple immune cells. (* represents P <0.05, ** represents P <0.01, and *** represents P <0.001).

We compared and analyzed the immune cell infiltration of LUAD samples and the control group with the ssGSEA algorithm. The graph shows the distribution of 28 immunocytes in two datasets, GSE63459 and GSE17634 (**Figure 9A**). Shown according to its results, the CD4+T cells, CD8+T cells, natural killer (NK) cells, and Bcell in LUAD samples were higher than that in normal samples, and this result indicates that these cells play a significant role in the progression of LUAD (**Figure 9B**). According to the correlation analysis of HUB genes and immune cell infiltration, most of these HUB genes showed a positive correlation with immune infiltrating cells, such as macrophage, CD4 + T cell, CD8 + T cell, and NK cell. CD8 + T cell exerts antitumor effects in a wide range of cancers. It should be noted that OCIAD2 and PARP1 genes are negatively associated with numerous immune cells. This fits with their results leading to the poor prognosis associated with patients with LUAD (**Figure 9C**).

### 3.8 Enrichment analysis of GSEA immune signature gene sets

**Figure 10:**
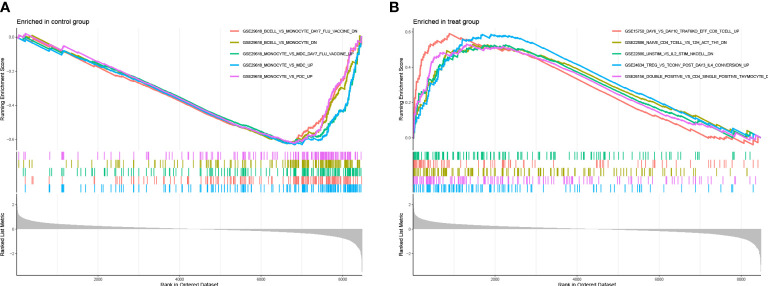
**(A, B)** plots represent the enrichment map of the GSEA immune marker database for the experimental and control groups, respectively.

To make out that immunogenetics may exist during the progression of LUAD, we have used the immunologically signature gene set in the MsigDB database as a reference standard for DEGS GSEA. A total of 819 gene sets were significantly enriched (|normalized enrichment score (NES)|> 1; FDR Q value <0.05). These genes were mainly concentrated in CD8 T cells, NK Cells, CD4+T cells, monocytes, and regulatory T cells (**Supplementary Table S1**). Based on the above findings, it appears that many immune genes play an essential role in LUAD progression (**Figure 10**).

### 3.9 Detection of mRNA levels of HUB genes by RT-qPCR

**Figure 11:**
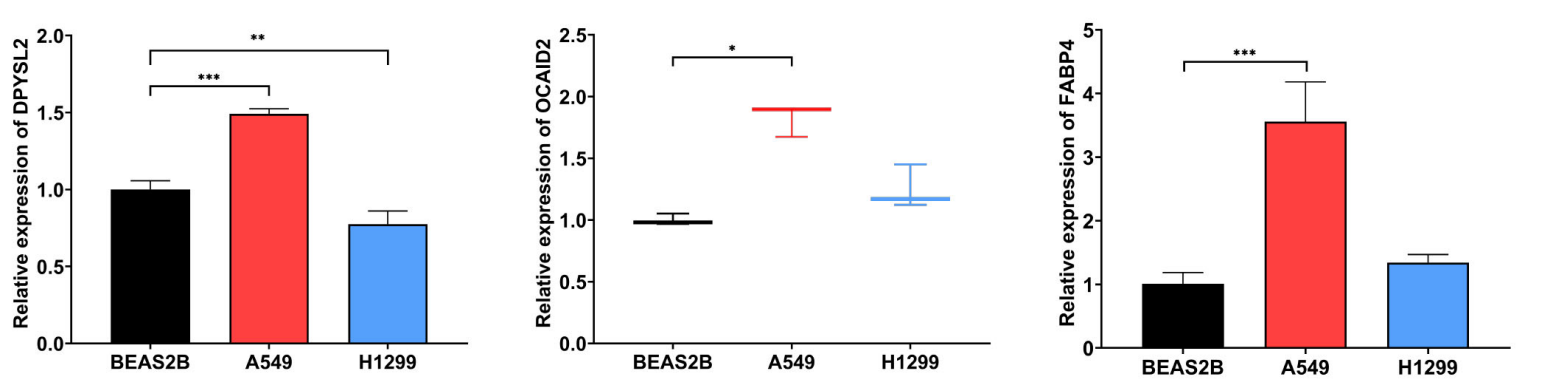
The mRNA levels of DPYSL2, OCIAD2, and FABP 4 were measured in human normal lung epithelial cell lines (BEAS-2B) and LUAD cell lines (A549 and H1299), respectively. (* represents P <0.05, ** represents P <0.01, and *** represents P <0.001).

After statistical analysis of the PCR results, we found that DPYSL2 (p <0.001), OCIAD2 (p <0.05), and FABP4 (p <0.001) had higher expression in A549 compared to BEAS-2B, showing a statistically significant difference. The expression content of DPYSL2 was lower in H1299 cells compared to t BEAS-2B (p <0.01). Although the expression difference of FABP 4 and OCIAD2 genes in H1299 was not statistically significant, they both showed a trend to increase (**Figure 11**). These experimental results can better support our study. Nevertheless, the results may require further study with a larger sample size.

## 4 Discussion

Using high-throughput microarray technology is a more efficient and accurate bioinformatics method when finding and screening key genes related to the mechanism of cancer development. This technology can also help us diagnose and treat diseases and help us in the design of new drugs. DEG is primarily enriched in leukocyte activation and production, alveolar development, the development of angiogenesis as well as certain immune responses, which are related to the mechanism of LUAD development (17). Analysis of the KEGG showed that DEGs were primarily enriched in immune-related pathways (IL-17 signaling pathway, TNF signal channel, The TGF signaling pathway, PPAR signaling pathway, and other pathways). The cytokine IL-17 can mediate a variety of physiological effects (18, 19). IL-17 is produced primarily by both innate and adaptive immune cells, whose main role is to exert its immune regulatory function by promoting the production of various proinflammatory cytokines and chemokines (IL-6, IL-23, transforming growth factor- β, tumor necrosis factor, etc.), leading to the accumulation of neutrophils and macrophages at the site of inflammation (20–22). IL-17 can stimulate lung tumor growth by promoting angiogenesis and proliferative activation (23, 24). IL-17 in the immune microenvironment can also induce lung cancer metastasis and spread (25). It has also been shown that treatment with a neutralizing anti-IL-17A antibody can reduce the angiogenesis of the tumor as well as reduce the inflammatory response, thereby reducing the growth of lung cancer progression (24, 26). IL-17 is overexpressed in a variety of lung cancer types. During the development of LUAD, the recruitment of numerous neutrophils by IL-17 leads to sustained inflammatory damage (27). All point to a strong link between IL-17 with LUAD progression and prognosis, and these studies are in good agreement with the DEGS enrichment results indicating that there are indeed genes within DEGS that are important in LUAD development.

Traditional DEG-based screening approaches are only capable of local analysis of datasets, which can easily cause the omission and loss of core genes. WGCNA can help us to systematically construct individual biological interaction network maps that can help us to identify core genes that are highly associated with disease prognosis (28, 29). Therefore, we used WGCNA to search for genes highly associated with LUAD and crossed the present results to previous DEGS to obtain highly related and differential genes. After screening these genes by LASSO analysis, the next 12 HUB genes were identified: ADAMTS8, CD36, DPYSL2, FABP4, FGFR4, HBA2, OCIAD2, PARP1, PLEKHH2, STX11, TCF21, TNNC1. These 12 key genes showed distinct differences in expression levels in LUAD samples and healthy samples. Notably, Of these, only OCIAD2 and PARP1 were found to be significantly highly expressed in tissues from LUAD samples, whereas the remaining 10 genes showed higher levels of expression in the control groups.

ADAMTS8 comes from integrins and metalloproteinases of the thrombospondin motif, and some studies show that ADAMTS8 is closely associated with vascular endothelial growth factor A (VEGFA), and some studies have found that ADAMTS8 expression in lung cancer is very low (30, 31). DPYSL2 is extremely highly associated with breast cancer, which can be expressed in breast cancer cells through axonal guidance. We can also use DPYSL2 to inhibit breast cancer progression and metastasis by inducing reversal. At the same time, numerous studies have demonstrated that phosphorylation of DPYSL2 and DPYSL2 is associated with drug resistance and tumor metastasis (32, 33). OCIAD2 belongs to the ovarian cancer immune response antigen (OCIA) domain family, which consists of 154 amino acids. It fulfills its role in tumor metastasis by promoting STAT3 activation and cell migration. And OCIAD2 is also highly expressed in lung adenocarcinoma but also ovarian mucinous tumors (33, 34). PARP1 is the central enzyme for cellular PAR production and a major target for polyadenosine diphosphate ribosylation during DNA damage. Upon DNA strand breaks, PARP1 performs DNA repair by covalently connecting the ADP-ribose moiety to the acceptor site of some amino acids on itself and other proteins (35, 36). Transcription factor 21 (TCF21) belongs to the class bHLHII superfamily of transcription factors and is expressed in various tissues and organs, it’s not only related to different biological processes, such as the development of the reproductive system (support cell differentiation and sex determination), respiratory system, spleen development, it also involved in regulating RNA polymerase to transcription process and so on (37, 38). CD36 is a membrane glycoprotein, as well as a scavenger receptor, which is found in a wide variety of cells. CD36 plays a major role in regulating atherosclerosis *via* a variety of pathways (39, 40).

The above studies we performed showed that DEGS is inextricably linked with inflammatory response, immune response, various cytokines, chemokines, and IL-17 factors. In this study, the infiltration of 28 immunocytes in the immune microenvironment of LUAD samples was investigated by the ssGSEA algorithm. The results showed that CD4 + T cells, CD4 + T cells, CD8 + T cells, natural killer (NK) cells, and Bcell were more infiltrated in LUAD samples than in normal samples. All of these cells are important in LUAD progression (24, 41, 42). However, following correlation analysis of HUB genes and infiltrating immune cells, in our study, most of these HUB genes were found to positively correlate with immune-infiltrating cells such as Macrophage, CD4 + T cells, CD8 + T cells, and NK cells. While CD8 + T cell has extensive anticancer effects (43). Macrophages play an immune role in a variety of tumors (lung cancer, breast cancer, liver cancer, etc.) (44, 45). Notably, OCIAD2 and PARP 1, which are inversely related to many immune cells, coincide with the results that these two genes are associated with the poor prognosis of lung adenocarcinoma. IL-17 mainly originates from Th17 cells, while Th17 cells mainly originate from CD4 + T cells, and high-level expression of IL-17 leads to inflammatory damage of inflammatory cells like neutrophils and leads to tumor vascular growth, both of which lead to the progression and metastasis of lung tumors. The enrichment of Tregs (regulatory T cell) is correlated with the occurrence, progression, metastasis, and prognosis of various malignancies, including lung cancer (46). Whereas the transcription factor Foxp 3 is the main regulator of Treg cell development and inhibitory activity, and this transcription factor is closely related to autoimmune diseases and a stable immune environment (47). In addition to producing plasma cells involved in the pathological process of LUAD, B cells can produce various cytokines that stimulate Tcell activation, thereby reducing the anti-inflammatory properties of regulatory Tcell and promoting the proliferation and differentiation of effector T cells. The above findings indicate that T cell homeostasis in the immune microenvironment is related to the occurrence, development, and prognosis of LUAD (48). Finally, to investigate the possible immune mechanisms during the development of LUAD, we used the immunological marker gene set in the MsigDB database as a reference for DSGS GSEA and found that activated CD8 T cells, NK Cells, CD4+T cells, monocytes, and regulatory T cells had enhanced expression in DEGS. This suggests that LUAD progression may be linked to the activation of T lymphocytes, monocytes, B lymphocytes, and various cytokines produced by themselves or by their interactions. These studies suggest that these HUB genes may have a potential relationship with the development of LUAD.

To conclude, we selected turquoise by WGCNA and LASSO regression analysis, combined with multiple bioinformatic analyses, and ultimately selected the 12 HUB genes with the highest correlation to LUAD (ADAMTS8, CD36, DPYSL2, FABP4, FGFR4, HBA 2, OCIAD2, PARP1, PLEKHH2, STX11, TCF21, TNNC1), and we analyzed and verified the functions of these genes in the present study. The results of this study will provide initial insights and novel insights into the underlying immunomodulatory mechanisms of LUAD. We will further investigate and explore the more sensitive and specific diagnostic markers of LUAD to provide new directions for LUAD diagnosis, treatment regimen, prognosis, and drug design.

## Data availability statement

The original contributions presented in the study are included in the article/**Supplementary Material**. Further inquiries can be directed to the corresponding author.

## Author contributions

AZ and DP designed this work. YZ performed the validation of the experiments. YF and SW integrated and analyzed the data. ZX, SS and XW wrote this manuscript. XG edited and revised the manuscript. All authors approved this manuscript.

## Supplementary material

Supplementary Figure 1 Prognostic relationships of 12 HUB genes and LUAD patients.

Supplementary Table 1 Enrichment analysis of GSEA immune signature gene sets
