# Supplementary figures and images for: Comprehensive analysis to identify a novel diagnostic marker of lung adenocarcinoma and its immune infiltration landscape
Source: Front Oncol. 2023 Jun 20;13:1199608. doi: 10.3389/fonc.2023.1199608 (PMC10319060; doi:10.3389/fonc.2023.1199608)

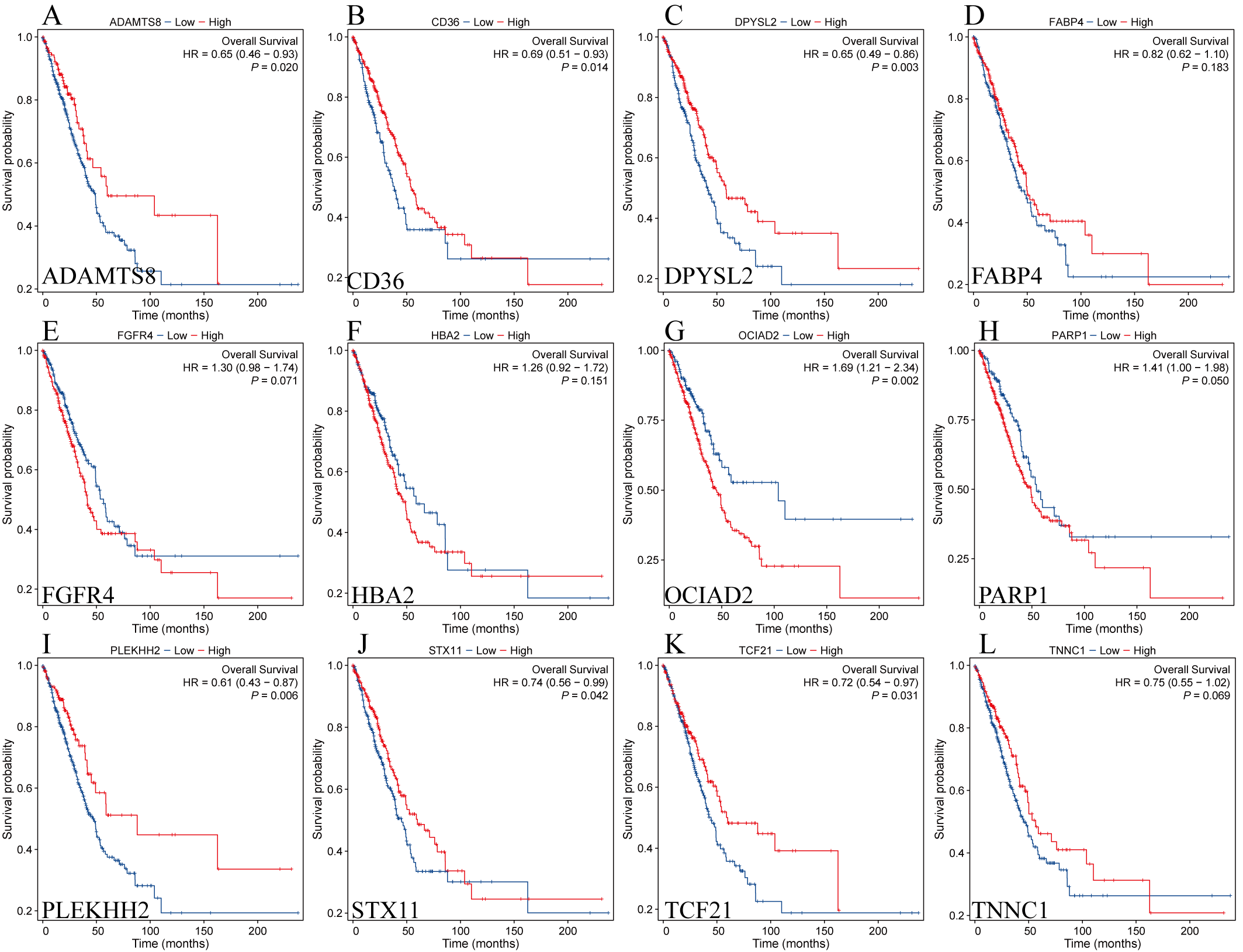

Supplement: Supplementary Figure 1 — Prognostic relationships of 12 HUB genes and LUAD patients. [file Image_1.tif]
